# *Ab initio* design of nanostructures for solar energy conversion: a case study on silicon nitride nanowire

**DOI:** 10.1186/1556-276X-9-531

**Published:** 2014-09-26

**Authors:** Hui Pan

**Affiliations:** 1 Institute of Applied Physics and Materials Engineering, Faculty of Science and Technology, University of Macau, Avenida da Universidade, Taipa, Macao SAR, People's Republic of China

**Keywords:** Silicon nitride nanowire, Solar energy harvesting, Doping, First-principles calculation

## Abstract

Design of novel materials for efficient solar energy conversion is critical to the development of green energy technology. In this work, we present a first-principles study on the design of nanostructures for solar energy harvesting on the basis of the density functional theory. We show that the indirect band structure of bulk silicon nitride is transferred to direct bandgap in nanowire. We find that intermediate bands can be created by doping, leading to enhancement of sunlight absorption. We further show that codoping not only reduces the bandgap and introduces intermediate bands but also enhances the solubility of dopants in silicon nitride nanowires due to reduced formation energy of substitution. Importantly, the codoped nanowire is ferromagnetic, leading to the improvement of carrier mobility. The silicon nitride nanowires with direct bandgap, intermediate bands, and ferromagnetism may be applicable to solar energy harvesting.

## Background

As one of the green energy sources, solar energy has been widely investigated to replace the old forms of depletable energy, such as coal and oil, which are limited on earth and detrimental to global climate. It needs, therefore, to develop reliable technologies to efficiently convert solar energy to other usable energy forms, such as electricity and chemical energy. A few technologies have been developed to harvest solar energy, including photovoltaic cells (PV; converting solar energy to electrical energy), photoelectrochemical cells (PEC; converting solar energy to chemical energy), and solar thermal systems (converting solar energy to thermal energy). In all of these technologies, the fundamental element, materials, plays a dominant role to maximally utilize the sunlight. For photovoltaic cells, the optimum bandgap for the solar cell material is a compromise between a bandgap wide enough so that not too many electrons are wasted and yet narrow enough so that enough photons can be absorbed to create electron–hole pairs [1]. For photoelectrochemical cells, the development of an efficient photocatalyst for water splitting requires (a) narrowing its bandgap that satisfies the visible light absorption and the band edge requirement of H_2_/H_2_O and O_2_/H_2_O levels [2,3] and (b) having high contacting surface area with the electrolyte to enhance the reaction and to increase the light absorption [4]. However, no semiconductor has a bandgap that can utilize the entire spectral distribution of sunlight. To enhance the light absorption efficiency, considerable effort has been conducted for the maximal absorption of sunlight, such as chemical doping [2-8], dye sensitization [9-11], material design [12-14], defect engineering [15-17], and structure engineering [18-20]. However, novel cell concepts are necessary for a huge increase in the efficiency. One of the concepts, hot-carrier solar cell, is to use semiconductor nanocrystals or quantum dots to capture all of the energy of hot carriers [21,22], where hot-carrier relaxation is only possible via slower multiphonon emission because of the quantum confinement-induced discretized band states in the nanostructures [23]. Another important concept is the intermediate band (IB) solar cell, consisting of an IB material situated between two conventional semiconductors, n- and p-types [24-26], where the IB material has a band inside the bandgap. A full electron transition from the valence band to the conduction band can be completed by means of two photons with energy below the bandgap, resulting in the increase in photocurrent. The IB can arise from the quantum confinement effects in quantum dots [27-29] or impurity states by doping bulk materials with a transition metal [30-33]. Recently, solar cells based on nanostructures have attracted considerable attention because of possible cost reduction and efficiency improvement [34-36]. Therefore, nanostructures possessing an intermediate band and quantum confinement effect may be able to enhance the efficiency and reduce the cost at the same time.

Silicon nitride (Si_3_N_4_) is a material of great technological interest in a number of applications, such as high-temperature electronics, because of its chemical inertness, high dielectric constant, large electronic gap, high resistance against radiation, and strong resistance against thermal shock [37]. Importantly, Si_3_N_4_ is a well-known antireflection coating material in the semiconductor industry to reduce the light reflection in Si-based solar cells [38]. Also, single-crystal Si_3_N_4_ nanowires on a Si substrate can be easily synthesized by chemical vapor deposition [39]. It is expected that an energy-harvesting cell based on Si_3_N_4_ nanowires and the present Si technology may make the Si_3_N_4_-based energy harvesting cell possibly produced on sustainable improved efficiency and cost reduction because of the easy integration of Si_3_N_4_ into the Si technology. In this work, we explore the electronic, magnetic, and optical properties of a Si_3_N_4_ nanowire for its possible application in solar energy conversion based on first-principles calculation. Our calculations predict that the Si_3_N_4_ nanowire is a direct-band semiconductor with reduced bandgap, and IBs can be created by doping with carbon and transition metals. We further show that anion-cation codoping can improve the solubility of a transition metal in Si_3_N_4_ and its crystallinity and enhance the magnetic moment. We further predict that the ferromagnetic Si_3_N_4_ nanowire with IB is more efficient for solar energy conversion.

## Methods

**Figure 1:**
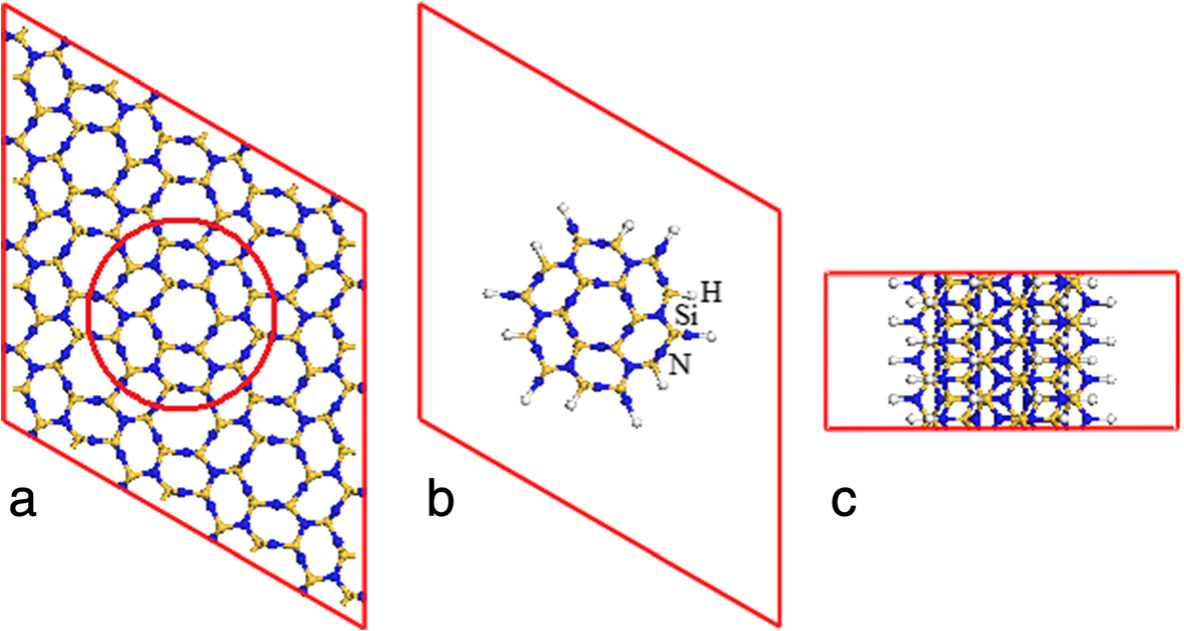
**Schematic drawings of the atomic structures of β-Si**_**3**_**N**_**4 **_**bulk (a) and nanowire: (b) top and (c) side views.** The red circle in **(a)** indicates the model of the nanowire in **(b)** and **(c)** created from the bulk. The yellow, blue, and white dots are Si, N, and H, respectively.

To investigate the electronic, optical, and magnetic properties of the Si_3_N_4_ nanowire, first-principles calculations are carried out based on the density functional theory (DFT) and the Perdew-Burke-Ernzerhof generalized gradient approximation (PBE-GGA) [40-42]. The Vienna *Ab initio* Simulation Package (VASP) incorporated with the projector augmented wave (PAW) scheme is used [43,44]. The nanowire is created along the β-Si_3_N_4_ [001] direction (Figure 1). An energy cutoff of 450 eV is used for the plane wave expansion of the electronic wave function. The Monkhorst-Pack scheme is used to generate special *k* points with a 1 × 1 × 3 grid [45]. Good convergence is obtained with these parameters. The total energy is converged to 2.0 × 10^-5^ eV/atom while the Hellmann-Feynman force is smaller than 5.0 × 10^-2^ eV/Å in the optimized structure. A large hexagonal supercell with a shortest wall-wall distance of 10 Å in the plane perpendicular to the wire axis and four unit cells parallel to the wire axis is used to avoid interactions between the wire and its images in neighboring cells and between dopants. The nanowire has 96 nitrogen and 72 silicon atoms.

To study the optical property of the nanowire, we calculate the loss function, which is a direct probe of the collective excitation of the system under consideration. The imaginary part of the dielectric constant is calculated from:

(1) $$\begin{array}{l} \epsilon_{2}\left(q\to O_{\overset{⌢}{u}},\hbar \omega \right)=\frac{2e^{2}\pi }{\Omega \epsilon_{0}}\sum_{k,v,c}|<\Psi_{k}^{c}|\overset{⌢}{u}\cdot r|\Psi_{k}^{v}\\ \;\;>|{}^{2}\delta \left(E_{k}^{c}-E_{k}^{v}-E\right) \end{array}$$

where $\overset{⌢}{u}$ is the vector defining the polarization of the incident electric field. This expression is similar to Fermi's golden rule for time-dependent perturbations, and *ϵ*_2_(*ω*) can be thought of as detailing the real transitions between occupied and unoccupied electronic states. The real part, *ϵ*_1_(*ω*), is obtained by the Kramers-Kronig relation. The loss function is calculated using Im(-1/*ϵ*(*ω*)) at zero momentum transfer from the macroscopic dielectric function *ϵ*(*ω*) (*ϵ*(*ω*) = *ϵ*_1_(*ω*) + *iϵ*_2_(*ω*)) for a periodic system.

## Results and discussion

### β-Si_3_N_4_ bulk

**Figure 2:**
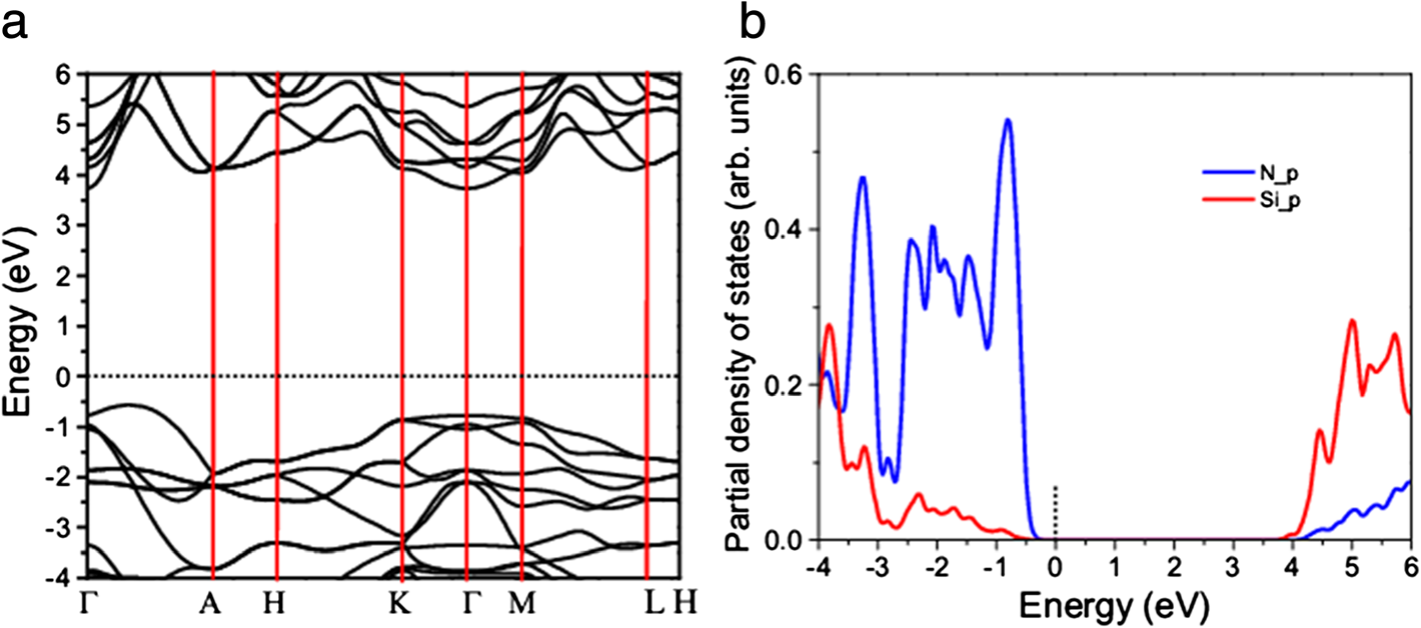
**Calculated band structure (a) and partial density of states (b) of β-Si**_**3**_**N**_**4 **_**bulk.**

The periodic unit cell of β-Si_3_N_4_ is optimized first to obtain the lattice parameters. The lattice constants of the optimized structure (*a =* 7.613 Å, *c* = 2.910 Å) within PBE-GGA are in good agreement with the experimental values (*a =* 7.608 Å, *c* = 2.909 Å) [37]. β-Si_3_N_4_ is an indirect-bandgap semiconductor with the valence band top (VBT) at one third along the Γ-A axis and the conduction band bottom (CBB) at the Γ point (Figure 2a). The calculated bandgap is 4.25 eV within PBE-GGA (Figure 2), which is consistent with the reported data (4.2 eV) [46], but less than the experimental value (5.3 eV) due to the underestimation of the bandgap by density functional theory. The analysis on the partial density of states (PDOS) reveals that the VBT states are mainly dominated by N_*p* electrons, while Si_*p* electrons mainly contribute to the CBB states (Figure 2b).

### β-Si_3_N_4_ nanowire

**Figure 3:**
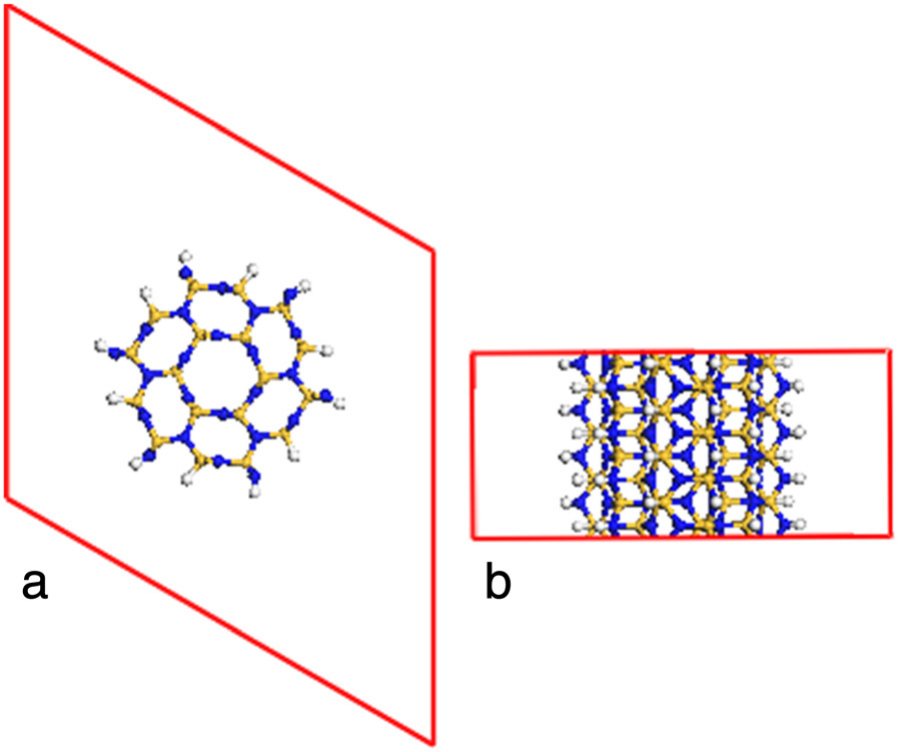
**The optimized atomic structures of β-Si**_**3**_**N**_**4 **_**nanowire (a) top and (b) side views.**

The β-Si_3_N_4_ nanowire is modeled by cutting the bulk supercell along the [001] direction (Figure 1a). The surface atoms are passivated by hydrogen atoms (totally 48 H atoms in the supercell) (Figure 1b,c). The structure of the optimized Si_3_N_4_ nanowire almost keeps unchanged, except the hydrogen atoms at the surface (Figure 3). The Si-N bond length is expanded by less than 1%. The Si-H and N-H bond lengths are 1.489 and 1.019 Å, respectively.

**Figure 4:**
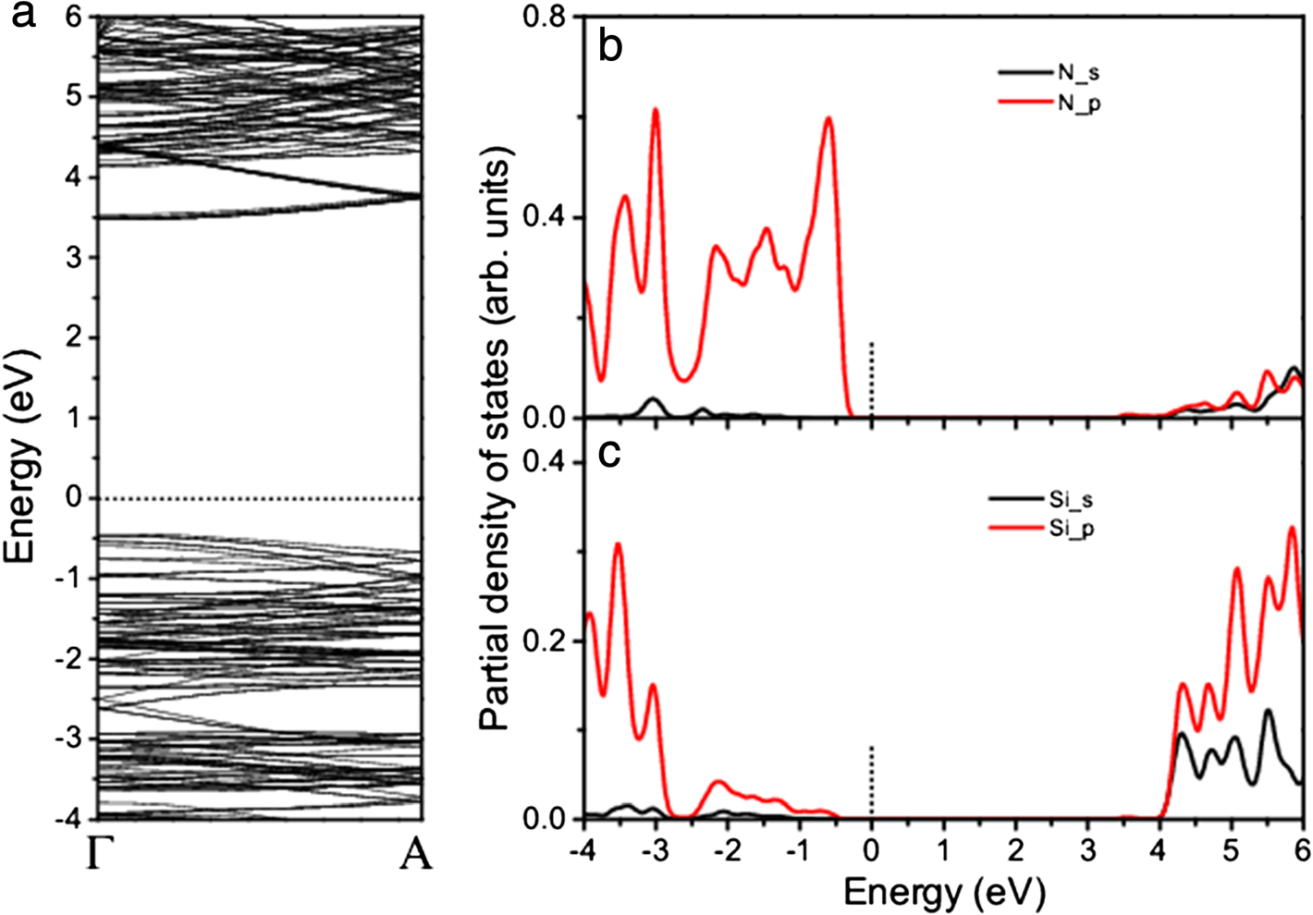
**Calculated band structure (a) and PDOS for N (b) and Si (c) of β-Si**_**3**_**N**_**4 **_**nanowire.**

The calculated electronic property of the nanowire shows a notable feature in its band structure (Figure 4). The nanowire is a direct-bandgap semiconductor with both the VBT and CBB at the Γ point. Compared with those in the band structure of β-Si_3_N_4_ bulk along the Γ-A axis, the valence band top states in that of the nanowire are pushed up, especially the states at the Γ point, leading to almost flat bands from the Γ point to one third of the Γ-A axis (VBT in the band structure of β-Si_3_N_4_ bulk) (Figures 2a and 4a) and the occurrence of VBT at the Γ point. The change of the VBT states is attributed to the confinement of charge carriers which results in the quasi-continuous energy bands of the bulk semiconductors becoming discretized. The VBT states are still dominated by N_*p* electrons (Figure 4b). The CBB remains at the Γ point with the parts of the CBB states in the nanowire pulled down. In contrast to the β-Si_3_N_4_ bulk, the *p* electrons from both N and Si contribute to the CBB states in the nanowire (Figure 4b,c). The up-shift of VBT and down-shift of CBB lead to the reduction of bandgap (3.92 eV). The direct band structure in the nanowire may enhance the conversion efficiency due to the improvement of carrier transportation. However, the bandgap of the nanowire is still too larger to be efficient of the sunlight absorption. It is necessary to engineer the bandgap to improve the absorption.

### Single-element-doped β-Si_3_N_4_ nanowire

One of the important ways to engineer the bandgap of semiconductors is to create intermediate bands within the gap by doping. Three elements, oxygen, carbon, and chromium, are studied because the contamination of oxygen and carbon easily occurs in the growth of Si_3_N_4_ and chromium is the most possible element for the creation of IB in the bandgap [31,32]. The doping is modeled by substituting a cation atom with a chromium atom or an anion atom with an oxygen or carbon atom inside the nanowire. As an indication of the doping possibility and stability, the doping formation energy is estimated from [47]:

(2) $$E_{f}=E_{\mathrm{tot}}\left(\mathrm{NW}+\mathrm{doping}\right)-E_{\mathrm{tot}}\left(\mathrm{NW}\right)-\mu_{\mathrm{dopant}}+\mu_{\mathrm{host}}$$

where *E*_tot_(NW *+* doping) and *E*_tot_(NW) are the total energies of the β-Si_3_N_4_ nanowire (NW) with and without doping, respectively. *μ*_dopant_ and *μ*_host_ are the energies of dopants (O, C, Cr, CrO, or CrC) and host atoms (Si, N, or SiN), respectively. $\mu_{O}=\frac{1}{2}\mu \left(O_{2}\right)$, $\mu_{N}=\frac{1}{2}\mu \left(N_{2}\right)$, *μ*_Cr_ = *μ*(Cr_Bulk_), *μ*_Si_ = *μ*(Si_Bulk_) and *μ*_C_ = *μ*(graphite). The formation energies of single-element doping (Table 1) indicate that the Cr atoms prefer to take the cation positions (Si sites), while the O and C atoms substitute the anion positions (N sites) more easily, because of the relatively lower formation energies. The formation energy for oxygen substituting nitrogen is only 0.75 eV, indicating that the doping of oxygen is much easier than that of other elements. The calculated bond lengths for the stable doping states are 1.799 Å for the O-Si bond, 1.826 Å for the C-Si bond, and 1.845 Å for the Cr-N bond in O-, C-, and Cr-doped nanowires, respectively, which are larger than the Si-N bond length (1.741 Å). The least change in the structure of the O-doped nanowire is consistent with lower doping energy.

**Table 1:** **Formation energy for the various doping configurations in the β-Si**_**3**_**N**_**4 **_**nanowire**

	**O-s-Si**	**O-s-N**	**C-s-Si**	**C-s-N**	**Cr-s-Si**	**Cr-s-N**
*E*_f_ (eV)	10.25	0.75	5.11	3.62	3.81	8.50

For example, O-s-Si means that O substitutes Si.

**Figure 5:**
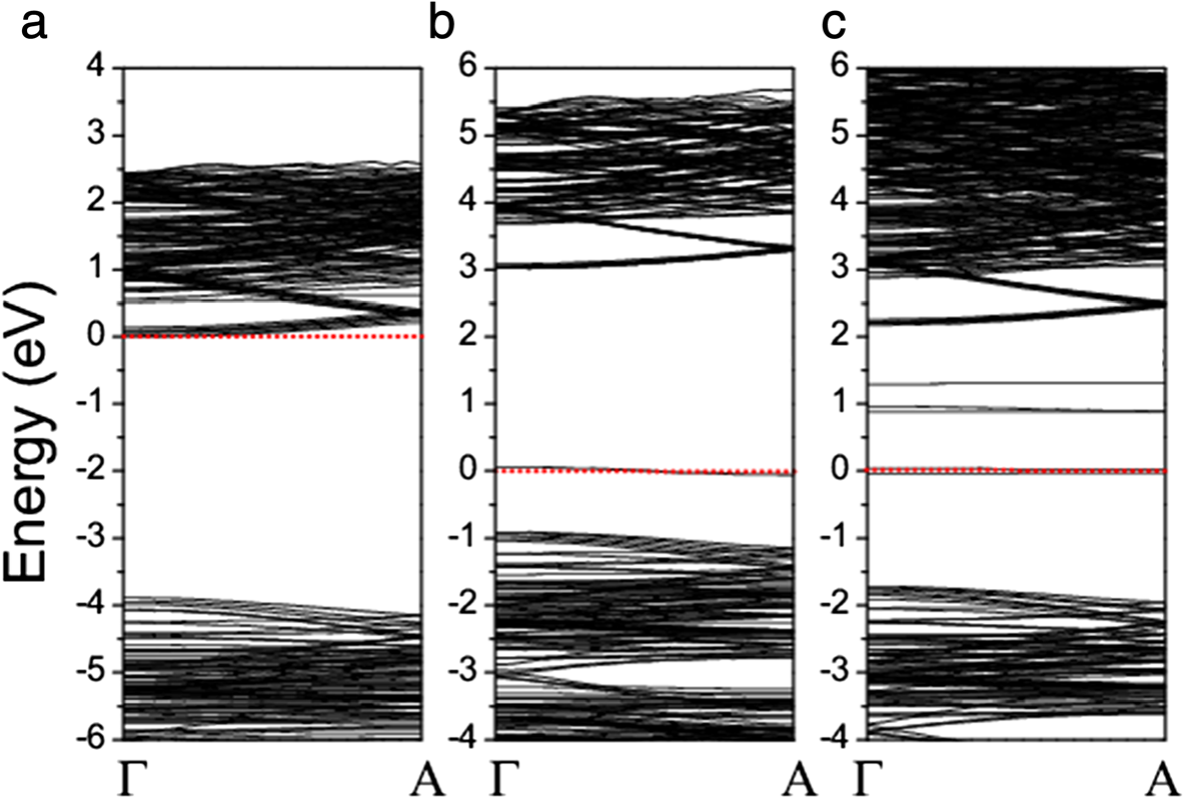
**Calculated spin-unpolarized band structures of β-Si**_**3**_**N**_**4 **_**nanowires doped with (a) O, (b) C, and (c) Cr.**

**Figure 6:**
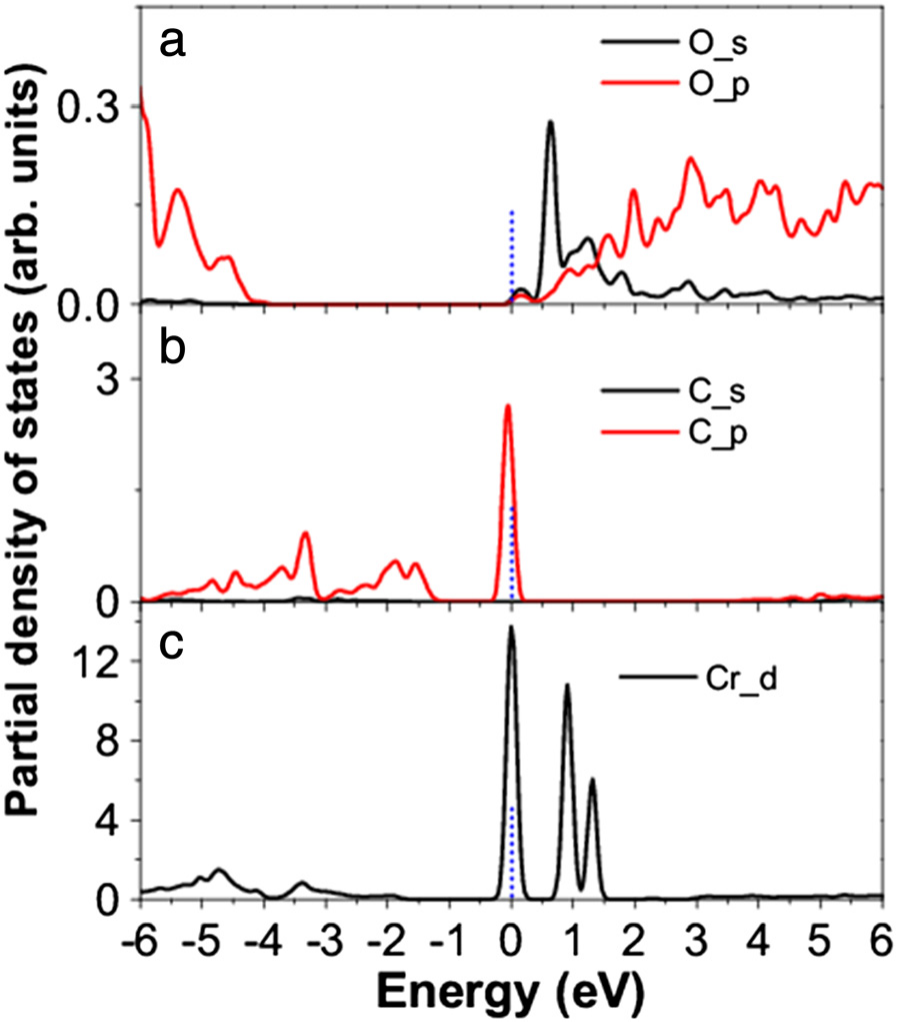
**Calculated spin-unpolarized PDOS of β-Si**_**3**_**N**_**4 **_**nanowires doped with (a) O, (b) C, and (c) Cr.**

The calculated band structure shows that the O-doped β-Si_3_N_4_ nanowire is an n-type semiconductor with a direct bandgap of 3.89 eV at the Γ point (Figure 5a). The PDOS analysis shows that the *s* and *p* electrons are also involved in the CBB states (Figure 6a). The band structure of the O-doped nanowire is similar to that of the undoped nanowire, except that the Fermi level is within the conduction band because of the contributed electrons from oxygen, indicating that O doping cannot efficiently narrow the bandgap of the nanowire. An intermediate level with less dispersion is formed by C doping, crossing the Fermi level (Figure 5b), which is dominated by the C_*p* electrons (Figure 6b). The gaps between the IB and VBT or CBB are 0.96 and 2.96 eV, respectively. Three IBs are formed by Cr doping, which separate the bandgap into four regions with the gaps of 1.68, 0.92, 0.42, and 0.88 eV from the VBT to CBB, respectively (Figure 5c). The impurity states are mainly attributed to the Cr_*d* electrons (Figure 6c). The Cr_*d* electrons should form a quasi-continuous impurity band in β-Si_3_N_4_ bulk, which are discretized in the nanowire due to the quantum confinement effect. The creation of IB by doping, especially Cr doping, should improve the sunlight absorption of the nanowire.

**Figure 7:**
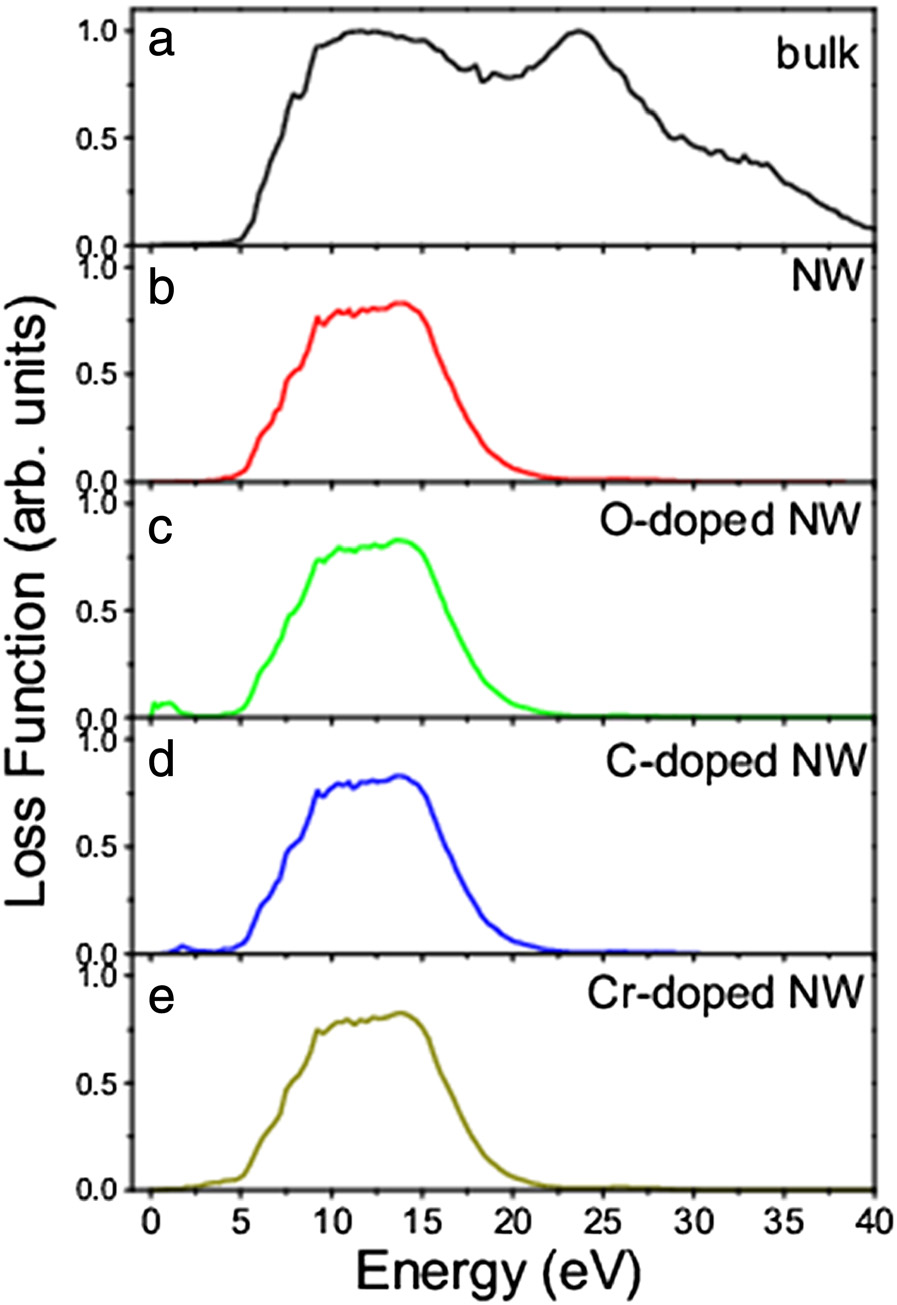
**Calculated loss functions of β-Si**_**3**_**N**_**4 **_**bulk (a), nanowire (b), and nanowires doped with (c) O, (d) C, and (e) Cr.**

The calculated loss functions of the β-Si_3_N_4_ nanowires clearly demonstrate the improvement of the light absorption, especially the C- and Cr-doped nanowires (Figure 7). The intercept in the loss function is the optical gap and related to the inter-band excitation. Comparing the loss functions of β-Si_3_N_4_ bulk and nanowire (Figure 7a,b), we see that the light absorption in the nanowire is redshifted because of the reduced bandgap. For the O-doped nanowire, an absorption peak starting from 0 eV is attributed to the electron excitation near the Fermi level because the O-doped nanowire is an n-type semiconductor (Figure 5a). The loss function of the C-doped β-Si_3_N_4_ nanowire is similar to that of the undoped one, except for a weak peak at 2.1 eV (Figure 7c), which is related to the inter-band excitation from VBT to IB or from IB to CBB (Figure 5b). For the Cr-doped nanowire, the loss function clearly shows that the excitation starts at 2.3 eV. From the loss functions of the doped nanowires, we can see that the IBs within the gap improve the absorption of the sunlight because of the reduced inter-bandgap (Figure 5c).

### Anion-cation-codoped β-Si_3_N_4_ nanowire

The codoping is realized by simultaneously substituting a cation atom with a chromium atom and an anion atom with an oxygen or carbon atom as a pair inside the nanowire. The calculated formation energies are 1.51 and 6.06 eV for CrO and CrC codoping, respectively. The formation energy of CrO codoping is much lower than that of Cr doping because of the electrostatic attraction of the two dopants with opposite charge states [31,32], which indicates that the Cr substitution can be greatly enhanced in the presence of O doping and results in the enhancement of solubility of Cr in the nanowire and improvement of crystallinity. However, the CrC codoping is unstable because of electrostatic repulsion between the two dopants, as indicated by the high formation energy, and will not be discussed. The Cr-N, Si-O, and Cr-O bond lengths in the CrO-codoped nanowire are 1.889, 1.703, and 1.996 Å, respectively.

**Figure 8:**
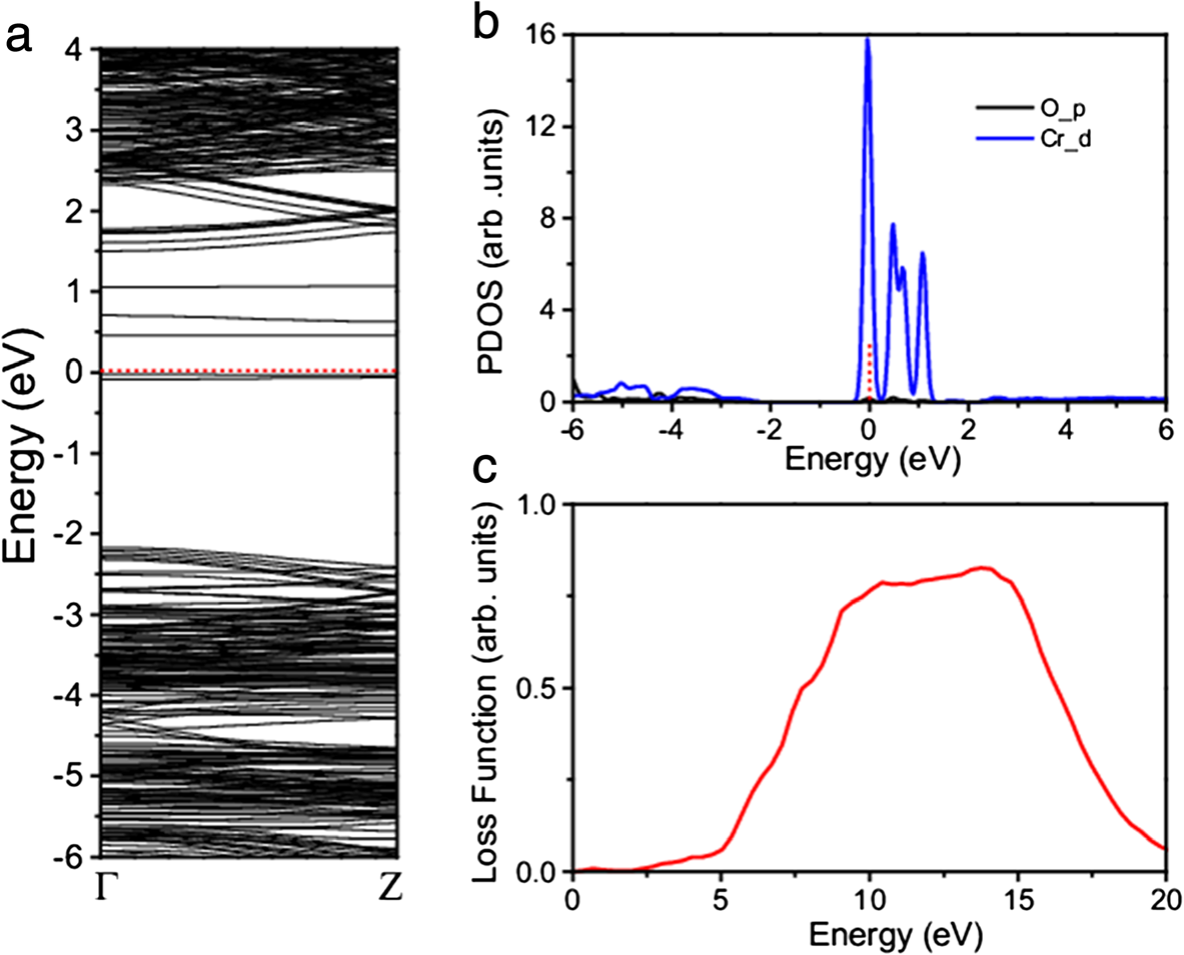
**Calculated spin-unpolarized band structure (a), PDOS (b), and loss function (c) of β-Si**_**3**_**N**_**4 **_**nanowire codoped with CrO.**

The calculated electronic and optical properties of the CrO-codoped β-Si_3_N_4_ nanowire show that four IBs are observable in the bandgap (Figure 8a), which are dominated by the Cr_*d* electrons (Figure 8b). The space between the VBT and the first IB is about 2.1 eV. Compared with those in the Cr-doped nanowire, the Cr_*d* electrons in the CrO-codoped nanowire are more discretized due to the quantum confinement effect and the strong coupling between the Cr_*d* and O_*p* electrons (Figure 8b). A similar situation occurs at the conduction band bottom, where more states become degenerate. Similar to Cr-doped nanowire, CrO-codoped nanowire also shows a excitation about at 2.3 eV (Figure 8c).

### Magnetic properties of Cr-doped and CrO-codoped β-Si_3_N_4_ nanowires

To investigate the magnetic properties of the doped nanowires, spin-polarized calculations are performed. To study the magnetic coupling between metal dopants, two neighboring Si atoms in the nanowire are substituted by two Cr atoms (Cr2) for the Cr-doped nanowire, and two neighboring Si atoms and one N atom bonded with them are replaced by two Cr atoms and one O atom (Cr2O) for the codoped nanowire. The calculated exchange energies, defined by the energy difference between the antiferromagnetic and ferromagnetic states (*E*_exch_ = *E*_AFM_ - *E*_FM_), are 135 and 293 meV for the Cr2-doped and Cr2O-codoped nanowires, respectively, indicating that the nanowires with doping are ferromagnetic. Importantly, the exchange energy of the codoped nanowire is much larger than that of the Cr-doped nanowire, leading to a much stable ferromagnetic state and higher transition Curie temperature of the codoped nanowire. The calculated magnetic moments per Cr atom are 1.85 and 2.29 *μ*_B_ for the Cr-doped and CrO-codoped nanowires, respectively. Clearly, the codoping enhances not only the exchange energy but also the magnetic moment.

**Figure 9:**
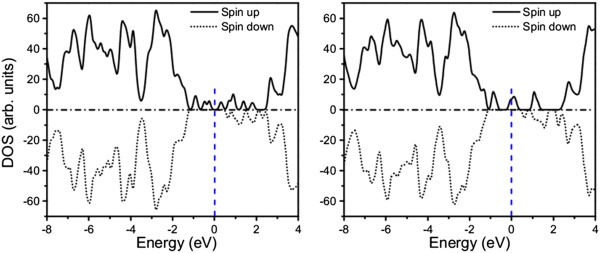
**Calculated spin-polarized total DOS of β-Si**_**3**_**N**_**4 **_**nanowires doped with (a) Cr and (b) CrO.**

**Figure 10:**
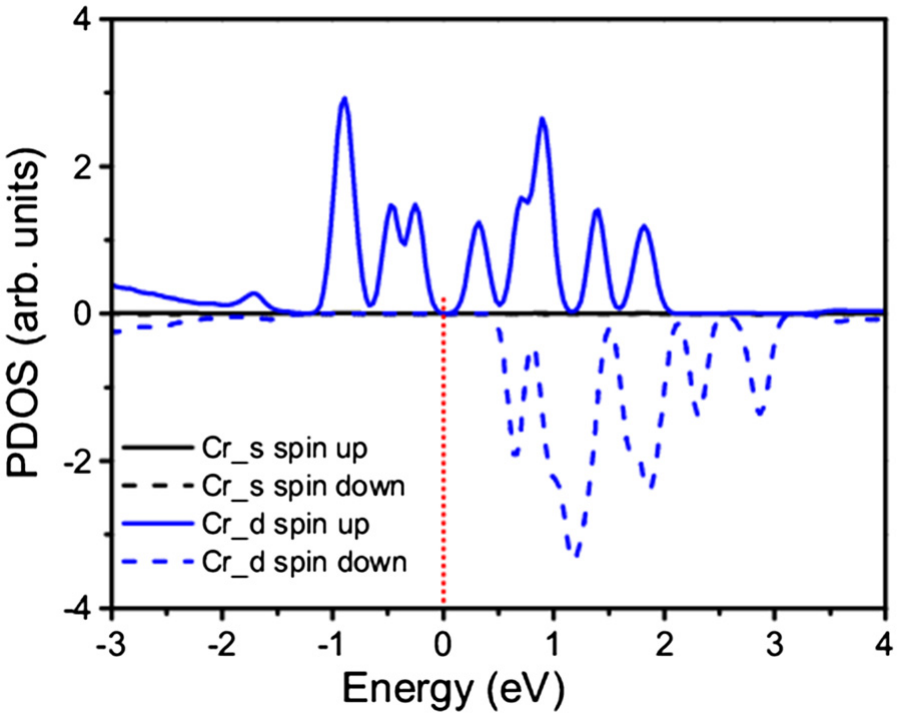
**Calculated spin-polarized PDOS of β-Si**_**3**_**N**_**4 **_**nanowire with Cr doping.**

**Figure 11:**
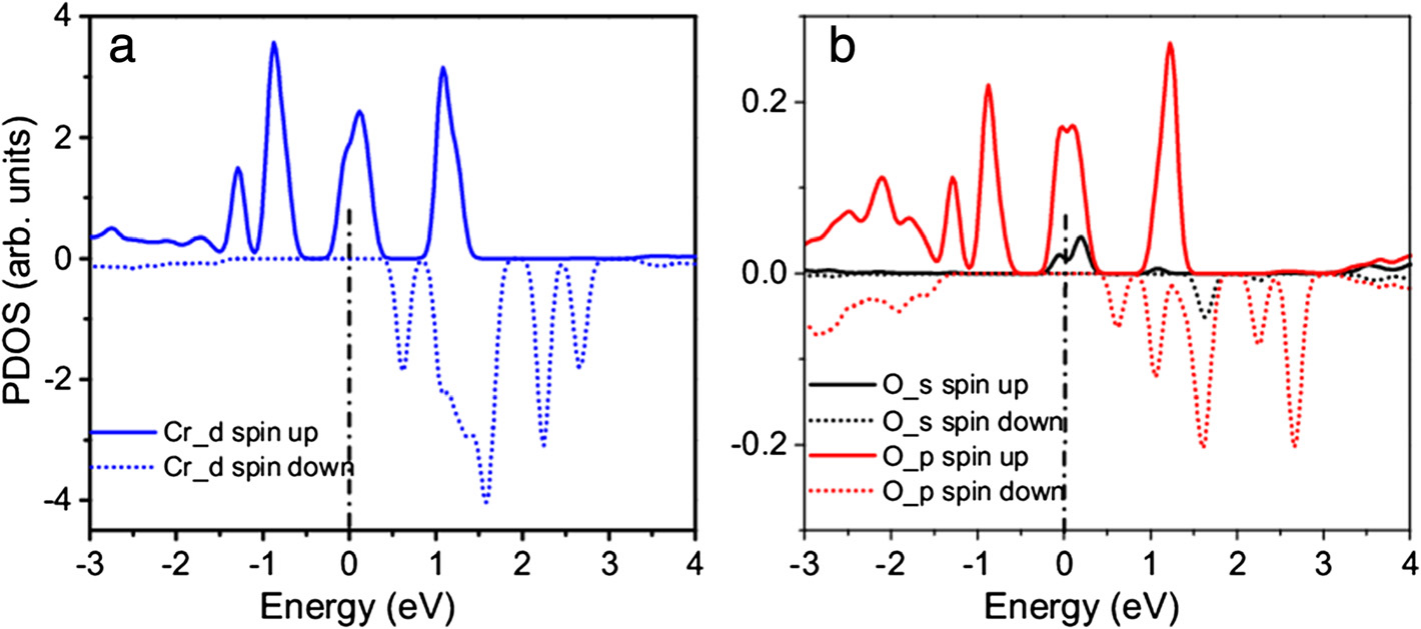
**Calculated spin-polarized PDOS of β-Si**_**3**_**N**_**4 **_**nanowire with CrO codoping for (a) Cr and (b) O.**

The calculated unsymmetrical spin-up and spin-down total density of states (DOS) confirm the ferromagnetism of the Cr-doped (Figure 9a) and CrO-codoped (Figure 9b) β-Si_3_N_4_ nanowires. All of these spin-polarized electrons are within the spin-up bands. For the Cr-doped nanowire, several impurity bands are observable and the Fermi level is in the gap between two impurity bands in the spin-up band (Figure 9a). The impurity states form three bands in the spin-up band, and the Fermi level is within the second impurity band when Cr codoped with oxygen (Figure 9b), indicating stronger polarization and enhancement of the magnetic moment. The PDOS analysis shows that the impurity states are mainly attributed to the spin-polarized Cr_*d* electrons for the Cr-doped nanowire (Figure 10), and for the CrO-codoped nanowire, the spin-polarized Cr_*d* and O_*p* electrons dominate the impurity bands (Figure 11). The Cr_*d* electrons strongly hybridized with the O_*p* electrons in the codoped nanowire, resulting in the stabilization of parallel spin alignment. The spin-polarized states within the bandgap (Figure 9) reveal that double exchange, which is stabilized by carrier (electrons) mediation, is the dominant coupling mechanism for the ferromagnetism in the Cr-doped and CrO-codoped nanowires [48-52]. That is, given the incomplete filling of bands, when the exchange splitting is bigger than the bandwidth, the band energy of the ferromagnetic state is lower than that of the antiferromagnetic state if a sufficient (usually rather small) number of holes (or electrons) exists [49]. The exchange splitting is bigger than the valence band (Figure 9), and there are a considerable number of carriers. We can therefore conclude that carrier-mediated double exchange is responsible for the observed ferromagnetism in the Cr-doped and CrO-codoped β-Si_3_N_4_ nanowires.

The calculated electronic structures (Figure 9) further reveal the enhancement of the sunlight absorption in the doped nanowires because of the formation of IBs within the bandgap. Importantly, the diluted magnetic semiconductors (DMSs) with IBs can have the desired optical properties and prevent radiative transition by taking the advantage of spin selection rules on IB transition [33]. The carrier mobility/lifetime is greatly enhanced because the spin degeneracy of the bands is lifted in DMSs and the unwanted recombinations are impeded by spin selection rules or by low occupancy of states involved in the allowed recombinations [53,54]. All of these advantages lead to the enhancement of the conversion efficiency of magnetic β-Si_3_N_4_ nanowires.

## Conclusions

In summary, a first-principles design of nanostructures is carried out to investigate their applications in solar energy harvesting. The calculated results show that the band structures of bulk materials can be engineered by reducing their size. Bulk Si_3_N_4_ is an indirect-bandgap semiconductor, while Si_3_N_4_ nanowire is a direct one. We show that the band structure of the nanowire can be further controlled by doping. Intermediate bands within its bandgap can be created by doping. The calculated optical property shows that the intermediate bands play an important role in the enhancement of visible light absorption. We also show that anion-cation codoping is easier than single-element doping because of the electrostatic attraction of the anion and cation. We further demonstrate that the ferromagnetic nanowire can be realized by codoping, where spin polarization can efficiently improve carrier mobility due to spin selection rules. The designed nanowire with a controllable band structure engineered by doping and size reduction shows efficient sunlight absorption and improved mobility and may find applications in solar energy harvesting.

## Competing interests

The author declares that he has no competing interests.
